# Dr. Tavernier's Treatise on the Treatment of Deformities of the Spine by the "Lever-Belt with Inclination Busk," without Extension Beds or Crutches; Containing a Relation of New Results Obtained, and the Class of Cases in Which the Belt May Be Safely Applied

**Published:** 1843-10

**Authors:** 


### Art. V.

*Dr. Tavernier's Treatise on the Treatment of Deformities of the Spine by the "Lever-belt with Inclination Busk," without Extension Beds or Crutches; containing a Relation of new results obtained, and the Class of Cases in which the Belt may be safely applied. Translated into English, with a Critical Analysis of this Division of Orthopædia, by* W. Brewer, m.d., London.—*London,* 1842. 8vo, pp. 43.

It was our original intention to place the present notice with the preceding, under one head, as having both reference to the same general subject. Further consideration, however, points out the propriety of keeping them distinct yet not far apart.

Dr. Tavernier's pamphlet is an inflated eulogium of the "Lever-belt with inclination busk." This instrument was invented by one Hossard, a French quack, whose name, by the way, is but once and incidentally mentioned throughout the entire treatise. Dr. Brewer, the translator, seems, however, to imagine that Dr. Tavernier is the inventor of this machine, for he prefixes to his translation a French dedication to that gentleman, in which he asks, "À qui pourrais-je dédier mon travail mieux qu'à vous-même? *à vous* dont *la belle Découverte* a bientôt frappé mon attention;" but if Dr. Brewer conceives that the homage of a dedication should be paid to the author of the "belle découverte," it is to Hossard the quack that he should have rendered it. This mistake on the part of the translator is the stronger, as Dr. Tavernier, though chary of mentioning Hossard by name, by no means claims the invention for himself; on the contrary, he expressly says that "a stranger to the medical body happened to light upon it by one of those sudden inspirations," &c. (p. 7;) and goes on to tell how the inspired stranger "boldly knocked at the door of the learned societies, holding his orthopedic bandage in his hand." (pp. 7-8.) Dr. Tavernier is only the apostle of the discovery, and Dr. Brewer is the missionary for the English district.

No one could ever suspect from the present pamphlet what the true history of the lever-belt really is; and as the facts of the case are not devoid of interest, we shall state them succinctly, especially as so doing will also enable us to appreciate Dr. Tavernier's treatise more connectedly and concisely than would otherwise be practicable.

Hossard, we believe in 1833, presented his lever-belt to the French Academy as a most efficient means of remedying spinal deformities, and a commission was appointed by that learned body to inquire into and report on its efficacy. Hossard presented several patients affected with lateral curvature of the spine to the commissioners, who were to inspect them again when the treatment was completed, and then draw their conclusion as to its results. Upwards of a year, however, elapsed without anything being heard of these patients; but during the period that the matter was thus sub judice, Hossard diligently advertised his "belt" as having been solemnly "approved of" by the Academy of Medicine, (Gazette Méd. de Paris, 1835, p. 123,) and caused handbills to that effect to be distributed on the Pont Neuf, and in the streets of Paris after the most Goss and Lamert fashion. Some members of the Academy having complained of this scandalous proceeding, Hossard, in 1835, again placed himself in communication with the commissioners, and the result was that M. Bricheteau at length, on the 8th Sept. 1835, (Gaz. Méd. 1835, p. 605,) reported to the Academy that Hossard had submitted to the examination of the commissioners three patients, two of whom laboured under the ordinary form of lateral curvature of the spine, while the third, named Janny Guery, was affected with a *single* and very considerable dorsal curvature; and that after a treatment of four and a half months, the last-mentioned patient and one of the former were perfectly straight, while the other was considerably improved. The commission consequently concluded—we shall here borrow Dr. Brewer's translation—"That the lever-belt, presented by M. Hossard, combats with much energy, and redresses as quickly the lateral curvatures of the vertebral column; and as to the application of the means, they do not appear to the members of the commission to subject the patient to any inconvenience during the treatment which they have observed." (p. 9.) *But that time and new experiments are still requisite to allow of all the advantages of the method being definitively appreciated.*" MM. Villermé, Moreau, Villeneuve, Roux, Double, himself one of the commissioners, and others, during the discussion on the Report, expressed their apprehensions that so much of those conclusions as were decidedly favorable would be turned to trade account by Hossard, but that the qualifying clause which we have underlined would be suppressed. The foresight of these gentlemen has been justified, for this clause is omitted by Dr. Tavernier, who, we should state, is, or at least was, medical consultant to Hossard's orthopedic establishment. This report we have said was read on the 8th Sept., and on the 15th of the same month, M. J. Guerin addressed a letter to the Academy, in which he stated, 1st, That the curvature was simulated in the case of Janny Guery, who was waiting-maid to Me. Hossard; and 2d, That the other two patients had been treated by Hossard for many months before they were presented to the commissioners, who were consequently deceived as to the time really occupied in the treatment, which, in point of fact, had been sixteen or seventeen months, instead of four months and thirteen days, and that in these cases, too, the amount of deformity that really existed had been collusively exaggerated. (Gaz. Méd. 1835, p. 606.) On the receipt of these allegations the Academy enlarged the original commission, and directed the matter to be investigated. Accordingly on the 12th Jan. (Gaz. Méd. 1836, p. 42,) and 2d Feb. 1836, (Gaz. Méd. p. 92,) M. Paul Dubois brought up two Reports in succession, both of which agreed in stating:

"1st. It is certain that as regards the first two patients, M. Hossard *knowingly deceived* the commissioners of the Academy, by representing them as not having been yet submitted to any treatment, whereas he had treated them during many months at Angers. 2d. As to the third patient, waiting-maid to Me. Hossard, the documents brought forward by M. Guerin to prove that she was not deformed before her journey to Paris, are contradicted by certificates produced by M. Hossard, which go to establish that Janny's deformity might have been overlooked at Angers, as she concealed it by the arrangement of her dress, etc. Consequently, the commission, while severely blaming M. Hossard for fraud respecting the two former patients, thinks the accusation is not so well proved (n'est pas aussi bien prouvée) as respects the third, and concludes that a copy of M. Bricheteau's first report may be transmitted to M. Hossard, but only on condition that there is annexed thereto a copy of this second report, containing the disapprobation of the Academy."

We do not enter into the subsequent proceedings, in the course of which a commission, consisting of MM. Breschet, Amussat, Orfila, and Cruveilhier expressed their conviction that the deformity of Janny Guery was simulated, because we conceive their conviction was founded on inadequate proof; and indeed it is tolerably clear that these learned academicians were unwittingly influenced by a desire to throw the shield of their protection over M. Guerin, against whom Hossard had instituted an action of libel for some strictures which exceeded the legitimate bounds of scientific discussion.

All these details are carefully avoided in Dr. Tavernier's treatise. He appeals to the approbation of the academy as conclusive proof of the value of the "lever-belt," and represents that approbation as having been unqualified. Thus he says: "When a scientific medical discovery hath won the public, and authentic approbation of a learned body, like that of the Académie Royale de Médecine......it can, resting on public notoriety, meet calmly the disdain; see even the reproach of some critiques, stirred up by any interest but that of science; and, in the assurance of success, pass judgment on those who are unable, or have not the knowledge to appreciate it," (p. 8;) and again, in speaking of the three patients reported on by the first commission, he says: "It is clear these experiments had every possible authenticity, and their importance was enhanced by the effect of *new examinations*, to which they were subjected, in *consequence of many long and warm academical discussions.*" (pp. 8-9.)

The brief but faithful abstract which we have given of these "long and warm academical discussions," will enable our readers to judge of Dr. Tavernier's candour in representing them as having enhanced the value of the evidence in favour of the lever-belt.

It is right to mention that a commission was also appointed by the Academy of Sciences to report on the efficacy of the "lever-belt," but this commission, we believe, never made any report on the subject; in this we may possibly be mistaken, but we may, at all events, assume that the report, if made, was not very favorable, as Dr. Tavernier, though he repeatedly alludes to the experiments performed before the commissioners of the Institute, makes no mention of their report. Dr. Tavernier assumes that these experiments are so much proof in favour of the lever-belt, because they were made in presence of those commissioners, but of course, in the absence of any report on them, they must be taken as Dr. Tavernier's assertions and nothing more.

We should not have devoted so much space to this pamphlet did we not entertain a strong conviction that the "lever-belt" is really a very valuable instrument in the treatment of lateral curvature of the spine; and it is precisely because we entertain this conviction that we thought it right to reduce Dr. Tavernier's testimony in its favour to its proper value, and to show, as we think we have done, that his evidence must be taken with very considerable limitation. Our experience of it, we must at once acknowledge, has been very limited; we do not happen to be professed orthopedists, and have applied it in but two cases, but in these its effects were most satisfactory. Having then this impression in favour of the machine, but being well aware how unwarrantable it is to put forward a positive conclusion respecting the value of *any* method of treatment, until it has gone through the ordeal of numerous trials in the hands of different practitioners, and believing that nothing can injure any invention more than trying to bolster it up by false facts, we have thought it our duty to examine those chiefly relied on by Dr. Tavernier, and trust we have placed our readers in a predicament to enable them to draw their own conclusion respecting them. We shall now endeavour to explain what the lever-belt is, and how it is to be applied, though we fear our description will not be perfectly intelligible without the assistance of a figure.

The apparatus consists of a broad leather belt, which fits round the pelvis, and has a kind of semicircular steel rack attached to it at a point corresponding to the sacrum. A steel bar or busk, which fits vertically into this rack, can be placed and firmly fixed in any requisite degree of lateral inclination. A broad leather strap is fastened to the belt in front, and is destined, after being brought obliquely round the body, to be fastened by two or more tongues to studs towards the upper part of the busk. The belt round the pelvis is steadied by a strap passed underneath the thigh, in order to render it a sufficiently fixed point of resistance.

To apply this instrument, suppose in a case of right lateral curvature, the belt is first fastened rather loosely round the pelvis, and the busk is inclined laterally towards the left shoulder, the degree of its inclination depending on the amount of the deformity. The strap, which is fastened to the belt in front, is now passed obliquely in front of the thorax and under the right arm, so as to pass over the ribs, which project in consequence of the spinal deformity. The length of this strap is so adjusted that it cannot reach the busk while the body is erect, and the trunk must therefore be bent to the left side, and while it is in that position the strap is fastened to the busk. The patient is now obliged to endeavour to throw the body to the right side, in order to restore the equilibrium; and, to use Dr. Tavernier's words, "by this movement, which that part of the spine situated above the strap can alone perform, the upper half of the arch, which the curved spine presents, finds itself of necessity brought back, in conformity to the axis of the body." (p. 19.)

We may quote the following explanation of the mode of its action:

"It is then, as we may see, by the combination of two actions, one mechanical, and produced by the apparatus, the other physiological, resulting from the play of the muscles, that the method of the lever with inclination acts on the bent spine. It is, in other words, working to obtain an opposite effect to that which we desire to destroy, and by the intervention of the same forces, the same immediate causes, but acting in a contrary direction, and with the view of effecting a cure, that this apparatus acts beneficially on the vertebral column." (p. 20.)

Dr. Tavernier, we are inclined to suspect, rather overrates the general applicability and efficiency of this instrument. He has a chapter on "the cases which contra-indicate the use of the belt, and those which require it." (pp. 10-11.) He seems to limit its inefficiency to cases in which the bones have become preternaturally hardened, or consolidated by bony anchylosis; in other cases, he says, "where applied to timely, art will triumph, and seem sometimes even to surpass the limits of the 'possible.'" (p. 11.) We should mention that he cites a considerable number of cases of cure, in addition to the three already so fully discussed: but we are sorry he puts those three on an equal footing of authenticity with the additional ones; he thereby throws some doubt over the whole series. This we regret, as it is calculated to excite a prejudice against an instrument which we think well worthy of being fully tried in those cases hitherto so very unmanageable in a lamentably large proportion. Some of the cases, too, are in themselves likely to excite a little incredulity. Thus we have the figure of the back of a young lady, aged fifteen, who suffered from a very considerable curvature for three or four years, the cure is represented to have been effected in *six weeks.* In most of the cases, however, from five to seven months were occupied in the treatment. In order to render the cure permanent, Dr. Tavernier recommends the use of "a simple ordinary corset, to which are differently adapted, according to the case, large ribands, which have for their object to bend the spine in the direction of the busk employed for the treatment."

From what we have said it appears that our judgment, quantum valeat, is decidedly favorable as to the value of the "lever-belt;" if we have spoken of its inventor as a quack, it is because he resorted to the practices of a quack; and if we have extended some share of blame to Dr. Tavernier, it is because we think he has exhibited some want of candour in particulars already pointed out and to which we do not wish to return. But this does not prevent us from candidly stating our favorable opinion respecting the apparatus, which we may just state, without any wish of thereby derogating from the merit of its inventor, bears a considerable resemblance to one already proposed by Delpech.

A few words respecting Dr. Brewer's part in the present publication. We rather suspect that he is not aware of the facts we have laid before our readers. He commiserates Dr. Tavernier as a most injured man, and tells him that "There is a thing, Harry, which thou hast often heard of, and it is known to many in our land by the name of pitch; this pitch, as ancient writers do report, doth defile, so our good brother had better let it alone." Now M. J. Guerin is, we suppose, Dr. Brewer's pitch, and so far as we know, it is Hossard and not Dr. Tavernier who hath been defiled by this pitch.

Although we cannot say much in favour of Dr. Brewer's "Analysis of Orthopædia," (which just comprises some four pages in very large type,) we are obliged to him for aiding in making known the "lever-belt" in this country, and shall be happy to meet him again, in print, if the subject-matter of his lucubrations be equally interesting; but we must be pardoned for suggesting to Dr. Brewer a more idiomatic study of his vernacular English, if he proposes to persevere in the functions of a translator. We suspect that our neighbours across the channel will deem Dr. Brewer's "Dédicace" not very classical French; and his own countrymen will not fail to accuse him of a tendency to Gallicise his mother-tongue. *Redress* is not the English for *redresser; grievous* is bad Saxon for *grave;* and "physiological travail" (p. 10) smacks of what Dr. Brewer perhaps would call *outre-manche* English. In the following extracts from Dr. Brewer's translation, the words are no doubt English, but the construction is we know not whence, and the meaning is we know not what; we give the punctuation exactly as we find it:

"Another set, demanding more, with less art, outdare, with more or less hardihood, the thousand ills of beds of extension, so dangerous when the object is really to extend the spine, so useless and unworthy the name, when, after the example of the orthopedists of our day, they are resorted to periodically and for mere popular consideration, their utility is bounded by the object of simply keeping the individual reclining on her back. (p. 7.) ........ In this case, reputed incurable, the changes very remarkable to be observed here, were great enough for the traces of the deformity, before so apparent and shocking, to be easily concealed." (p. 32.) ........ We call to witness this, not only the authority of experience, but even the luminaries of the medical science, who would boldly tell us their objections; as also the good sense of parents, to whose sight and handling we would not fear to present this apparatus, the simplicity of which admits the most inexperienced to render an account of its action. (p. 33.) ........ It is easy to see that in these cases the supposed pressure exerted on the chest and the abdomen produced no great ravages, and that it would be difficult indeed to ally the smallest doubt of injury to the principal functions with such results,—with positive proof of the most perfect nutrition," &c. (p. 35.)

---

